# Evaluation of macular and peripapillary vessel flow density in eyes with no known pathology using optical coherence tomography angiography

**DOI:** 10.1186/s40942-017-0080-0

**Published:** 2017-07-31

**Authors:** Muhammad Hassan, Mohammad Ali Sadiq, Muhammad Sohail Halim, Rubbia Afridi, Mohamed K. Soliman, Salman Sarwar, Aniruddha Agarwal, Diana V. Do, Quan Dong Nguyen, Yasir Jamal Sepah

**Affiliations:** 1 Department of Ophthalmology, Byers Eye Institute, Stanford University, Palo Alto, CA 94303 USA; 2 Ocular Imaging Research and Reading Center, Menlo Park, CA USA; 3 Post Graduate Institute of Medical Education and Research, Chandigarh, India

## Abstract

**Purpose:**

To assess normal vessel flow density (VFD) in macular and peripapillary regions of eyes with no known ocular pathology using optical coherence tomography angiography (OCTA).

**Methods:**

AngioVue (Optovue, Fremont, CA, USA) was used to capture OCTA images. A 3 × 3 mm grid and a 4.5 × 4.5 mm grid was used to scan parafoveal and peripapillary regions, respectively. ReVue software was utilized to measure VFD in five sectors within the inner two circles of ETDRS grid in macular region and correlated to retinal thickness of same sectors. At optic disc, VFD was calculated in six sectors based on Garway-Heath map. Area and morphology of foveal avascular zone (FAZ) was correlated with VFD in central 1 mm. The influence of myopia on mean VFD was also assessed.

**Results:**

Twenty-four eyes (mean age: 30 years) were analyzed. Mean VFD in macular sectors was 43.5 (±4.5) and 45.8 (±5.0) % in superficial and deep retinal plexuses, respectively. Mean VFD was significantly higher in deep retinal plexus compared to superficial retinal plexus in all sectors except central 1 mm (p < 0.05). Mean VFD in central 1 mm increases with an increase in central retinal thickness in both superficial and deep retinal plexuses (p < 0.001). Mean parafoveal VFD at level of both superficial and deep retinal plexuses decrease with an increase in spherical equivalent in myopics (p < 0.05). Mean VFD in myopics was found to be significantly lower in parafoveal region of deep retinal plexus (p < 0.05). Mean area of FAZ was 0.33 (±0.15) and 0.47 mm^2^ (±0.15) in superficial and deep retinal plexuses, respectively. Area of FAZ decreases with an increase in central 1 mm thickness and foveal VFD (p < 0.001).

**Conclusions:**

OCTA may be used to measure VFD in macular and peripapillary regions. Vessels in the parafoveal region are more densely packed in the deep retinal plexus leading to higher VFD compared to superficial plexus. Thicker retina in fovea translates into higher foveal VFD due to more compact arrangement of retinal layers and continuity of inner nuclear layer (INL). Myopia is associated with lower VFD in parafoveal region at level of deep retinal plexuses which may explain thinning of INL in myopics.

## Background

The per gram oxygen demand of the retina has been described as being more than that of the brain, especially in the macular region [1, 2]. Therefore, to maintain this high oxygen demand, a healthy and well-regulated circulation is of vital importance. Retinal microvasculature is the target of a variety of retinal diseases ranging from inflammatory to occlusive processes. Therefore, imaging of the retinal microvasculature is an important component of ophthalmic examination.

Histologically, there are four layers of vascular network in the retina [3, 4]. These include superficial retinal plexus (SRP), intermediate retinal plexus (IRP), deep retinal plexus (DRP) and radial peripapillary capillaries (RPC). These retinal plexuses are mainly responsible for providing nutrients to the inner half of the retina and have no role in nutrition of the outer retina including the photoreceptors [5]. The location of the SRP is at the level of ganglion cell layer. The IRP and DRP are located just above and below the level of the inner nuclear layers. In the macular region, there is a specialized area devoid of vessels called the foveal avascular zone (FAZ), present both at the levels of SRP and DRP [6]. The FAZ is surrounded by a ring of capillaries sharply demarcating the avascular area. In the peripapillary region, RPC occupy the retinal nerve fiber layer (RNFL).

Fluorescein angiography involves the injection of a fluorescent dye into the circulatory system of the patient and has been reported to only effectively image the SRP [7, 8]. It is not very effective in providing details of the deeper retinal plexuses and the RPC.

Optical Coherence Tomography Angiography (OCTA) is a novel technique for imaging blood vessels without the need for intravenous contrast administration. It relies upon intrinsic motion contrast present in the vascular network and hence precludes the need for an artificial dye. Additionally, OCTA allows adequate visualization of the vascular abnormalities in various retinal diseases which would not be possible to visualize using the traditional angiography due to leakage, pooling or staining of the dye [9]. Furthermore, it can image of all the retinal plexuses. Majority of the studies using OCTA have been qualitative and have described various abnormalities in the microvasculature including disruption of the vasculature in retinal plexuses secondary to diabetic retinopathy, age-related macular degeneration, vascular occlusive disorders and uveitis [10–14]. Additionally some studies have quantified the vessel flow density (VFD) in the retinal plexuses [15–17]. In our study, we utilized the standardized Early Treatment of Diabetic Retinopathy (ETDRS) grid to report the VFDs in superficial and deep retinal plexuses in the macular region and in six sectors based on the Garway-Heath map centered on the optic disc. Therefore, our study aims to evaluate this quantitative method of measuring VFD in the standard macular and peripapillary regions of normal subjects. This would provide basis for use of as a standard method to assess the extent of vessel loss in various ocular disorders in future. Hence, VFD can be utilized as a standard outcome measure to monitor these diseases and assess the effect of various therapies used for their management.

## Methods

The local Institutional Review Board approved the research on healthy volunteers. The study was conducted in compliance with the declaration of Helsinki, US Code of Federal Regulations Title-21, and the Harmonized Tripartite Guidelines for Good Clinical Practice (1996). A written informed consent was obtained from all participants of the study. 24 eyes of 12 normal subjects were included in the study. Inclusion criteria for the study consisted of healthy volunteers with no known systemic or ocular diseases, except refractive error.

### Optical coherence tomography angiography (OCTA)

AngioVue (Optovue, Fremont, CA, USA), was used to capture the OCTA images. Two OCTA volume scans (one horizontal and one vertical) were acquired to decrease motion artifacts and fixation changes. Split-spectrum amplitude-decorrelation angiography was used to detect flow and produce OCTA images and en face sections. A 3 × 3 mm grid centered on the fovea was chosen to scan the parafoveal region. A 4.5 × 4.5 mm grid centered on the optic disc was used to scan the peripapillary region.

**Fig. 1:**
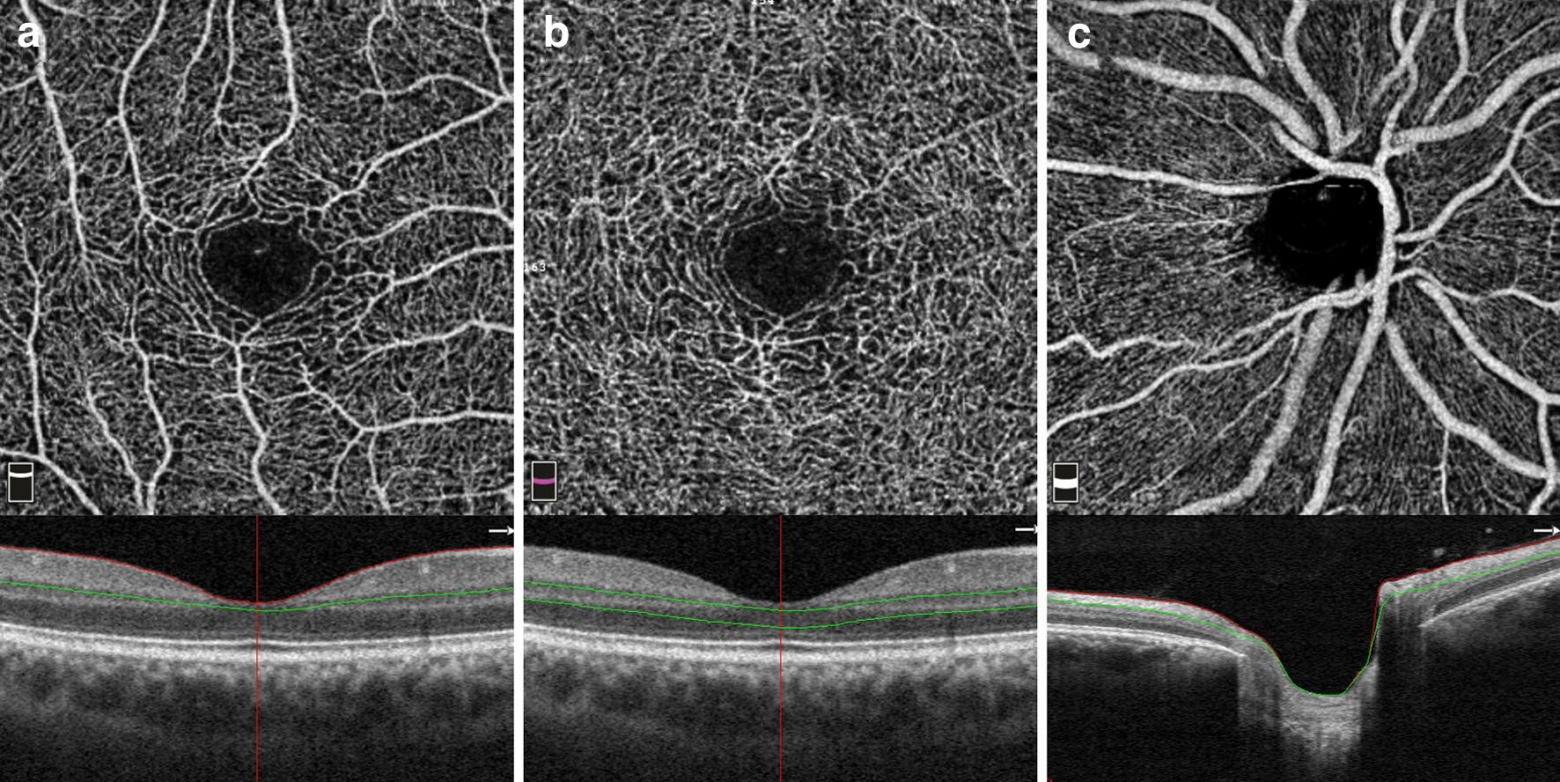
**a** Segmentation of superficial retinal plexus with an *inner boundary* at 3 mm beneath the internal limiting membrane (ILM) and an *outer boundary* set at 15 mm beneath the inner plexiform layer (IPL). **b** Segmentation of deep retinal plexus with an *inner boundary* 15 mm beneath the IPL and an *outer boundary* at 70 mm beneath the IPL at the level of inner nuclear layer. **c** Segmentation of peripapillary vascular plexus at the level of radial peripapillary capillaries (RPC) layer

ReVue software (Version: 2015.1.0.71) was used to segments the OCT scans and measure the VFD in the regions of interest (ROI). Each 3 × 3 mm parafoveal scan was automatically segmented with the following boundaries: the SRP was segmented with an inner boundary at 3 µm beneath the internal limiting membrane (ILM) and an outer boundary set at 15 µm beneath the inner plexiform layer (IPL) (Fig. 1a). The DRP was segmented with an inner boundary 15 µm beneath the IPL and an outer boundary at 70 µm beneath the IPL (Fig. 1b). In the peripapillary region the scan was segmented at the level of RPC layer (Fig. 1c). RPC layer is defined as the layer between the outer limit of retinal RNFL and the ILM. All scans were reviewed independently by two graders (MH and AS) to assess image quality and were repeated if the quality was found to be inadequate.

### Vessel flow density and area of foveal avascular zone

**Fig. 2:**
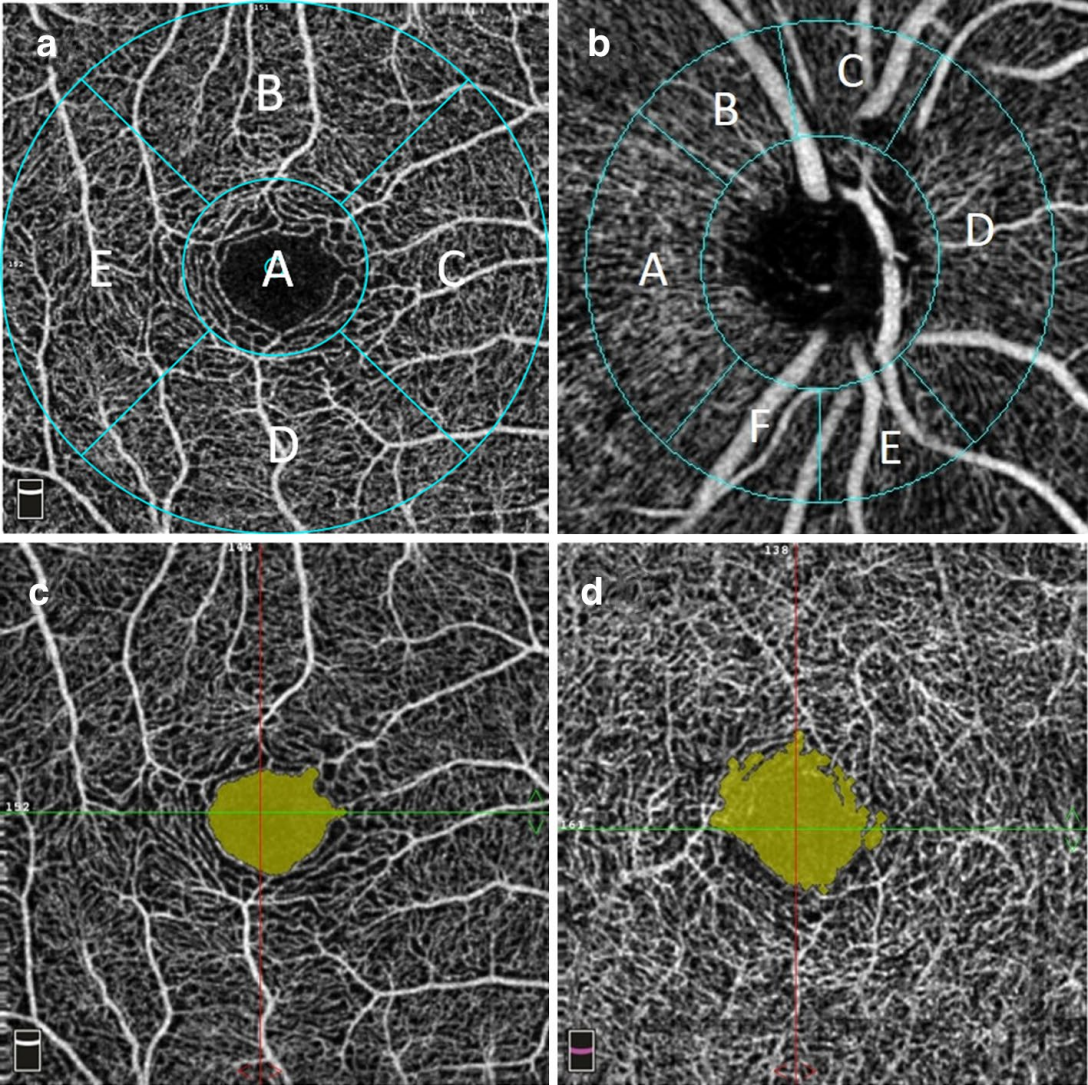
**a** Macular sectors for VFD analysis: *a* fovea, *b* superior, *c* nasal, *d* inferior, *e* temporal. **b** Peripapillary sectors for VFD analysis: *a* temporal, *b* superior temporal, *c* superior nasal, *d* nasal, *e* inferior nasal, *f* inferior temporal. **c** FAZ in superficial retinal plexus. **d** FAZ in deep retinal plexus

Vessel flow density (VFD) was measured by using the automated density measurement tool in the Revue software. In the macular region of interest (ROI), VFD was measured in five sectors within the inner 2 circles of the Early Treatment Diabetic Retinopathy Study (ETDRS) grid centered on the fovea (Fig. 2a). The diameter of the inner circle was 1 mm and the outer circle was 3.0 mm. The location of grid was manually corrected to center on the fovea if the default position calculated by the software was incorrect. The location of the foveal center was confirmed by cross referencing with the OCT scans associated with the OCTA image. The VFD measurements were noted at the level of both SRP and DRP.

At the optic disc, VFD was reported in six sectors based on the Garway-Heath map centered on the optic disc (Fig. 2b). The inner elliptical contour is obtained by fitting an ellipse to the disc margin shown on the OCT en face image. This was manually corrected in case the automated positing failed to do so. The ring width between the inner and outer elliptical contour was 0.75 mm in 4.5 mm × 4.5 mm scans.

The area of the FAZ was measured by using the non-flow measurement tool in the software. The area of the FAZ was identified on the angioflow image as area void of any vasculature. Figure 2c, d shows a sample of demarcated FAZ area in SRP and DRP.

### Optical coherence tomography

AngioVue (Optovue, Fremont, CA, USA), was used to capture standard cross sectional scans. The machine captures 1024 A-scans per B scans and 70,000 A-scans per second. The scan depth is up to 3-mm and the tissue axial resolution is 5 µm. Retinal map analysis protocol was used to obtain mean full retinal thickness measurements for each of the five sectors within the inner 2 circles of the ETDRS grid corresponding to sectors used in VFD measurement. Central retinal thickness (CRT) was defined as the average thickness of the central 1 mm diameter of the ETDRS grid.

### Spherical equivalent

Eyes having any degree of spherical and cylindrical error were classified as myopics. Mean spherical equivalent were calculated using the spherical diopter plus half of the cylindrical diopter power and utilized in this study for analysis. Hyperopic eyes were excluded from the analysis.

### Outcome measures

Mean VFDs in SRP and DRP were correlated with the retinal thicknesses in corresponding sectors on the grid in the macular region. The effect of demographic characteristics (age, gender and race) on mean VFD was also reported. Furthermore, the influence of myopia on the mean VFD was also assessed. The mean VFD in SRP and DRP was correlated with the spherical equivalents of the subjects. Mean VFD among myopics was also compared to the normal subjects. Area of FAZ was correlated with VFD in the central 1 mm and with the CRT.

### Statistical analysis

Stata V14.1 (Stata Corp, TX) was used to all statistical analysis. Mean and standard deviations in VFDs, were reported for all the sectors in the SRP and DRP in the macular region. Paired sample *t* test was used to compare mean VFD in SRP and DRP to account for eyes from the same patient. Multivariate linear regression analysis was performed to control for age, gender and race and document the correlation between VFD and the retinal thicknesses in the corresponding regions. *p* value of <0.05 was deemed significant.

## Results

Standard SD-OCT and OCTA were performed on 24 eyes of 12 healthy individuals with no known ocular and systemic illnesses except refractive errors. Mean age of the subjects was 30 years (range 24–49 years). Ten subjects (83.8%) were male. Majority of the subjects were Asian (75%). Other races included Caucasians (16.7%) and Africans (8.3%). All participants had best corrected visual acuity of 20/20. The average CRT was 252.8 ± 26.2 µm. Five subjects (10 eyes) in the study were myopics with mean spherical equivalent of −4.97 ± 1.97 D. None of the subjects in the study were hyperopic.

In the macular region, VFD was measured at both SRP and DRP. The SRP consists of large arterioles branching form the major vascular arcades and further divide into smaller arterioles which give rise to the superficial capillary network. DRP was noted to be a homogenous network of capillaries.

**Fig. 3:**
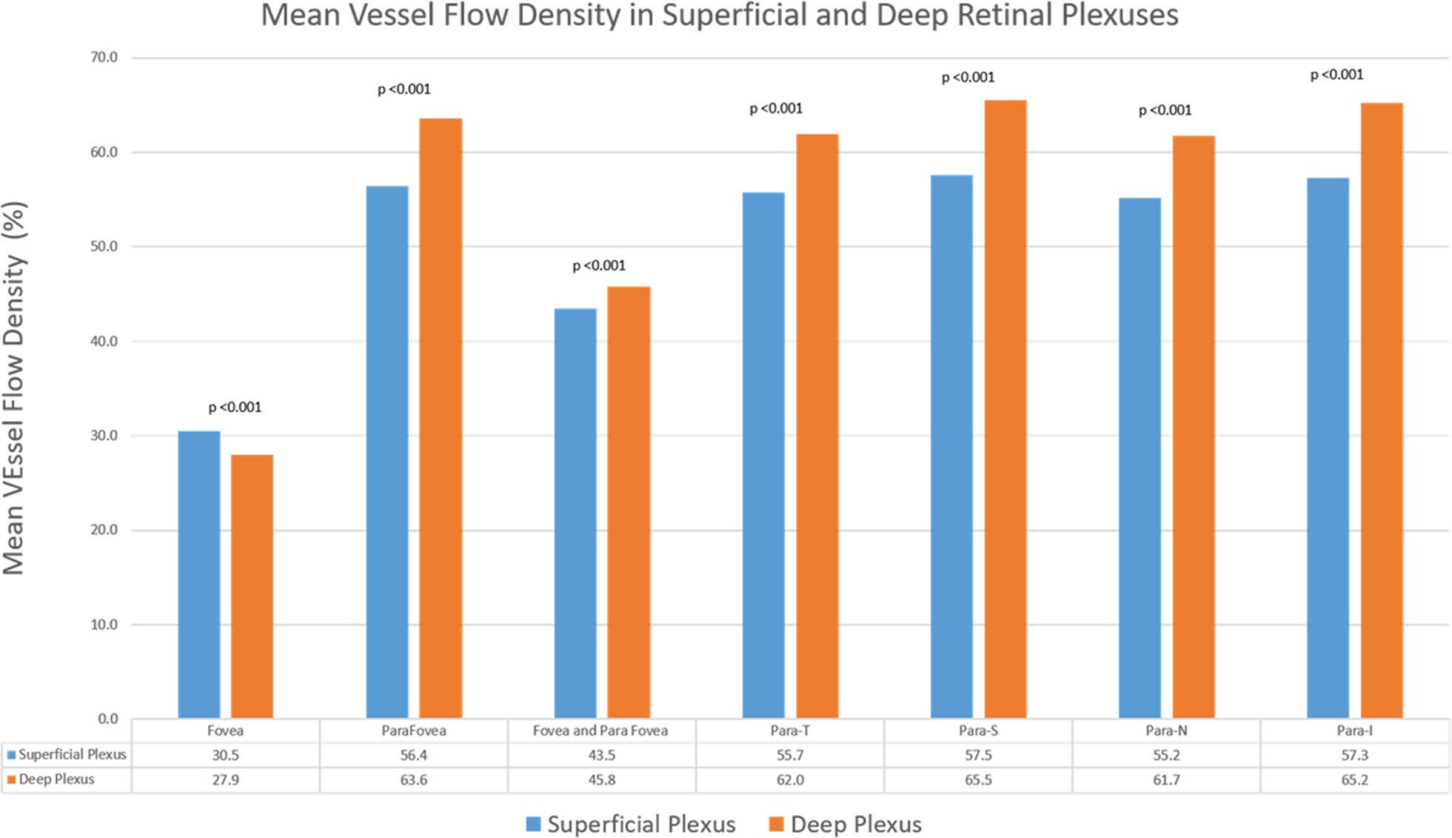
Comparison of VFD in superficial and deep retinal plexuses

The mean VFD in the 5 macular sectors at the level of the SRP was 43.5% (±4.5%), whereas the mean VFD at the level of the DRP was 45.8% (±5.0%). Figure 3 shows a comparison of mean VFD in the SRP and DRP in the five sectors of the grid. The mean difference in the mean VFDs between SRP and DRP was statistically significant in all sectors (p < 0.001). The mean VFDs in the DRP were significantly higher in all sectors except the foveal region where it was significantly lower.

A significant negative correlation of age with parafoveal VFD was seen at both the SRP and DRP [R = −0.26 (p = 0.006) and −0.182 (p = 0.008) respectively]. However, there was no correlation of age with foveal VFD.

**Fig. 4:**
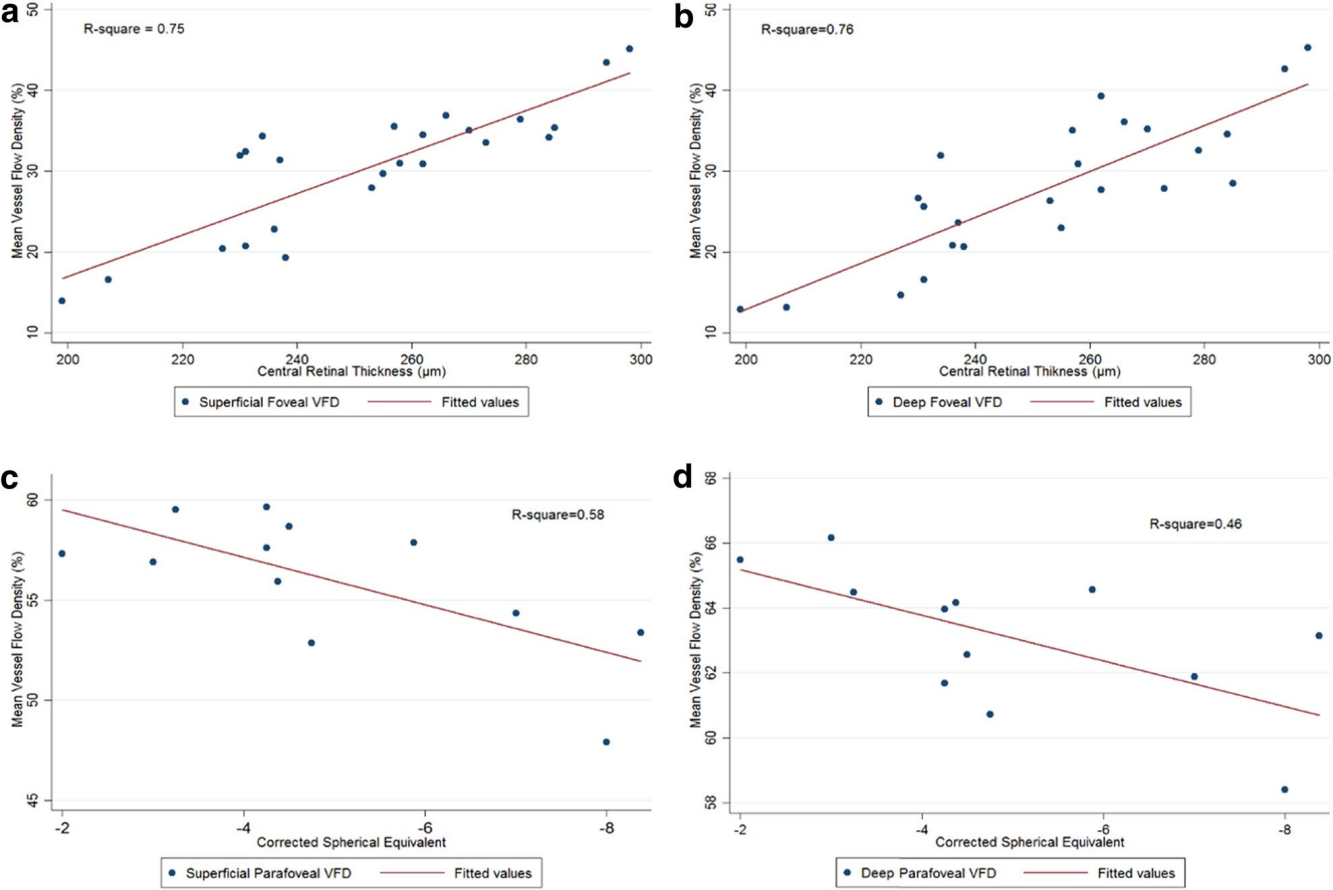
**a**, **b** *Scatter plots* depicting correlation between VFD and CRT in both superficial and deep retinal plexuses in the foveal region. **c**, **d** *Scatter plots* depicting correlation between VFD and spherical equivalent of subjects with myopia in both superficial and deep retinal plexuses in the parafoveal region. The *graphs* depict scatter of actual values around a predicted *line plot* showing strength of the prediction model

Table 1 outlines the results of regression analysis to correlate mean VFDs with mean retinal thicknesses in the macular sectors when controlled for age, gender and race. The mean VFD in the central 1 mm was strongly correlated with the CRT in both SRP and DRP [R = 0.29 (p < 0.001) and 0.29 (p < 0.001) respectively]. Figure 4a, b shows scatter plots showing correlation between CRT and VFD in the central 1 mm after controlling for age, race and gender.

**Table 1:** Correlation of central retinal thickness (CRT) with superficial retinal plexus VFD or deep retinal plexus VFD in the macular sectors when controlled for age, gender, and race

Sectors	Superficial retinal plexus					Deep retinal plexus				
	Mean retinal thickness µm (SD)	Mean vessel flow density % (SD)	Regression coefficient (R)	95% Confidence interval	*p* value	Mean retinal thickness µm (SD)	Mean vessel flow density % (SD	Regression coefficient (R)	95% Confidence interval	p value
*Macular sectors*										
Fovea	252.8 (26.2)	30.5 (7.4)	0.29	0.17 to 0.41	**<0.001**	252.8 (26.2)	28.0 (8.9)	0.29	0.17 to 0.41	**<0.001**
Parafoveal-superior	323.8 (13.8)	57.5 (2.8)	0.03	−0.07 to 0.14	0.50	323.8(13.8)	65.5 (2.2)	0.03	−0.06 to 0.11	0.54
Parafoveal-nasal	321.0 (16.7)	55.2 (3.5)	0.01	−0.09 to 0.12	0.78	321.0 (16.7)	61.7 (2.9)	0.09	0.02 to 0.16	**0.01**
Parafoveal-inferior	322.3 (14.3)	57.3 (4.1)	0.02	−0.12 to 0.16	0.74	322.3 (14.3)	65.2 (2.2)	0.07	−0.01 to 0.14	0.07
Parafoveal-temporal	311.4 (11.9)	55.7 (3.7)	0.03	−0.09 to 0.15	0.62	311.4 (11.9)	62.0 (3.0)	1.90	−0.20 to 3.99	0.07
Parafovea (combined)	319.5 (13.4)	56.1 (3.1)	0.02	− 0.08 to 0.13	0.69	319.5 (13.4)	63.8 (2.0)	3.61	0.34 to 6.89	**0.03**

p-value is significant (p < 0.05)

VFD of subjects with myopia (6 subjects) was correlated with the spherical equivalents of the subjects. A negative correlation was found between spherical equivalents and the mean VFD in the parafoveal region at both the SRP and DRP [R = −1.14 (p < 0.05) and −0.67 (p < 0.05) respectively] (Fig. 4c, d). There was no correlation found between spherical equivalent and VFD in the foveal region. Mean VFD in myopics was also compared to emmetropic subjects. Mean VFD in myopics was found to be significantly lower in the parafoveal region of the DRP (Table 2). This was more significant in the parafoveal nasal and temporal region (Table 2).

**Table 2:** Comparison of VFD between myopics and emmetropics at the level of superficial and deep retinal plexuses

Sectors	Emmotropics (n: 14)		Myopics (n: 10)		p value
	Mean	SD	Mean	SD	
*Superficial retinal plexus density*					
Fovea	28.99	2.68	32.70	0.83	0.27
Parafoveal-Superior	57.32	0.72	57.86	0.96	0.65
Parafoveal-Nasal	55.82	0.94	54.23	1.11	0.29
Parafoveal-Inferior	57.94	1.03	56.46	1.41	0.39
Parafoveal-Temporal	56.60	0.57	54.53	1.62	0.19
Parafovea (combined)	56.92	0.71	55.77	1.17	0.38
*Deep retinal plexus density*					
Fovea	27.89	3.05	28.03	1.22	0.97
Parafoveal-Superior	65.46	0.65	65.49	0.62	0.98
Parafoveal-Nasal	62.73	0.81	60.25	0.61	**0.03**
Parafoveal-Inferior	65.74	0.50	64.46	0.80	0.16
Parafoveal-Temporal	63.32	0.50	60.05	1.02	**0.0047**
Parafovea (combined)	64.31	0.45	62.56	0.62	**0.03**

p-value is significant (p < 0.05)

The area of FAZ was measured at the level of both SRP and DRP. In all the eyes the shape of FAZ was slightly irregular with considerable variation is the area between different individuals. Within subjects the mean size of FAZ was significantly larger and less well defined in DRP compared to the SRP [0.33 mm^2^ (±0.15) and 0.47 mm^2^ (±0.15) respectively, p < 0.05]. The area of FAZ controlled for age, gender and race, was negatively correlated with foveal VFD in SRP (regression coefficient = −45.8, p < 0.001). Similarly, negative correlation was also found between foveal VFD and area of FAZ controlled for age, gender and race in DRP (regression coefficient = −50.9, p < 0.001). The area of FAZ controlled for age, gender and race, in SRP and DRP was also negatively correlated with CRT [R = −96.5 (p < 0.001) and −117.2 (p < 0.001) respectively]. There was no statistically significant difference in area of FAZ between myopics and emmetropics. Additionally, linear regression demonstrated no significant correlation between area of FAZ and spherical equivalent in eyes with myopia.

The mean VFD in the peripapillary region was 60.4% (±5.0%). Table 3 shows mean values of peripapillary VFD in the six sectors based on the Garway-Heath map. Mean RNFL thickness in the peripapillary region was 102.5 µm (±10.7 µm). No significant correlation was found between the mean VFD in all the six sectors and the mean RNFL thickness in the corresponding areas.

**Table 3:** Correlation of RNFL thickness with VFD at level of superficial or deep retinal plexuses of retina in the peripapillary sectors when controlled for age, gender, and race

Sector	Mean vessel flow density (SD)	Mean RNFL thickness (SD)	Regression coefficient (R)	95% Confidence interval	p value
*Peripapillary sectors*					
Peripapillary region	60.4 (5.0)	102.5 (10.7)	0.08	−0.06 to 0.22	0.22
Temporal	62.7 (6.2)	76.3 (10.0)	0.10	−0.17 to 0.37	0.45
Superior-Temporal	63.3 (5.4)	139.9 (18.1)	−0.04	−0.16 to 0.08	0.52
Superior-Nasal	56.2 (8.9)	113.5 (16.0)	0.04	−0.16 to 0.25	0.67
Nasal	57.1 (3.5)	79.7 (12.4)	0.10	−0.01 to 0.22	0.08
Inferior-Nasal	61.3 (4.6)	114.7 (16.2)	−0.02	−0.13 to 0.10	0.78
Inferior-Temporal	61.9 (7.2)	139.7 (23.0)	0.11	−0.01 to 0.22	0.07

## Discussion

Retinal microvasculature is the target of a variety of retinal diseases ranging from inflammatory to occlusive in nature [18]. OCTA allows capturing of high-resolution images of the retinal microvasculature without the use of contrast.

In this study, we qualitatively and quantitatively assessed OCTA scans of healthy eyes in the macular and peripapillary regions. There was a significant correlation between retinal thickness and VFD in the foveal region. Spherical error in subjects with myopia was found to be negatively correlated to vessel flow density in the parafoveal region. In the peripapillary region, VFD was not found to correlate with RNFL thickness in sectors based on Garway-Heath map. Savastano et al. [8] effectively described in detail the retinal vascular morphology in healthy eyes as assessed by the OCTA. Similar to our study they were able to differentiate and describe morphology of two separate vascular plexuses.

**Fig. 5:**
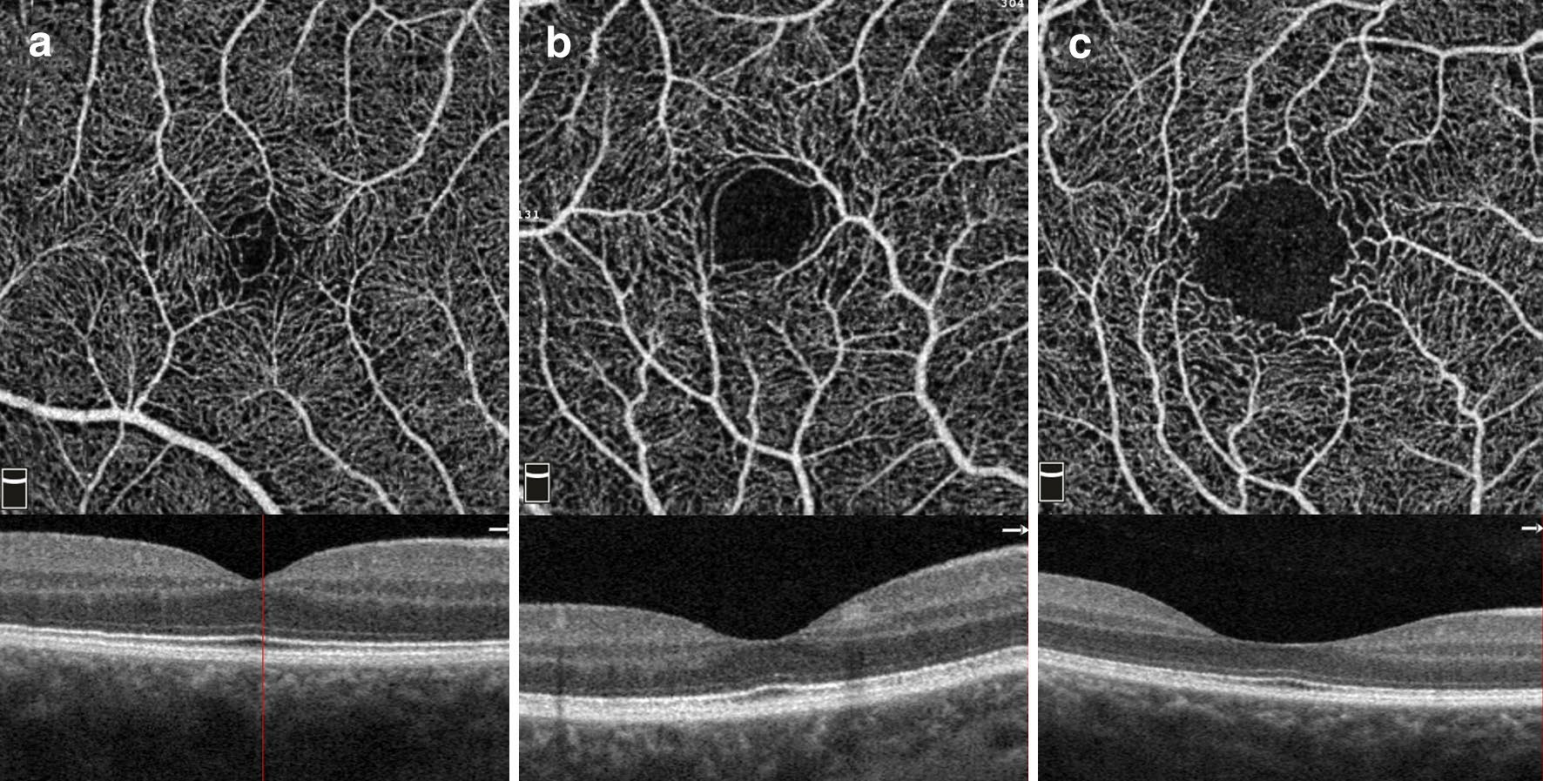
Correlation between greater CRT and greater VFD in central 1 mm and smaller area of FAZ in 3 healthy subjects. **a**– **c** OCTA of the macula (*above*) with the corresponding OCTA scans at level of superficial retinal plexuses (*below*). **a** CRT of 298 mm with high VFD of 45.2% and small FAZ area of 0.105 mm^2^. **b** CRT of 234 mm with lower VFD of 34.3% and larger FAZ area of 0.304 mm^2^. **c** CRT of 207 mm with lowest VFD of 16.6% and largest FAZ area of 0.629 mm^2^. In the thickest fovea **(a)**, there is continuity of inner nuclear layer and ganglion cell layer over the foveolar center. In the thinnest fovea **(c)** there is a large cleavage of the inner retinal layers at the foveal center. From the thickest to the thinnest fovea, an increasing VFD is noted

With the advent of the ability to image the retinal vasculature at different levels, it was important to find a way to quantify the presence of the vessels. Shahlaee et al. [16] utilized automated thresholding of the Image J software version 1.49 (National Institutes of Health, Bethesda, Maryland, USA) to assess the VFD in the foveal and parafoveal region. In this study, mean VFD reported in the parafoveal region was slightly lower than our study both at the level of SRP and DRP (45.96 and 51.57% respectively) compared to our study. This difference may be attributed to a slightly different algorithm used in our study. The thresholding algorithm converts the images of the vascular network into binary images and defines vessels as pixels above a certain threshold level. Thus, smaller capillaries which might fall below the threshold are not counted in the density measurement. Gadde et al. [15] utilized an automated local fraction dimension method to quantify VFD in OCTA. Similar to our findings, they found significant difference in VFD between SRP and DRP. In contrast to our study they reported that mean VFD in the peripapillary area was not affected by age. In another study swept source OCT optical microangiography was used to report the VFD in the macular region [17]. In this study the VFD at the level of SRP was reported to be higher (67.3%) compared to the DRP (34.5%). This result is in contrast to our study where the mean VFD is significantly higher in DRP (63.6%) compared to SRP (56%). The difference can be accounted for by the use of the semiautomatic color selection tool of GNU Image Manipulation Program for vessel selection by Kuehlewein et al. Since vessels in the DRP are more densely packed, the image manipulation software was unable to resolve between closely packed networks of capillaries. Both of these studies utilize the margin of FAZ to define the central circle, which is then subsequently excluded from the density calculations. In contrast the grid used in our study has a standard 1 mm central circle. We noted that the VFD in this central 1 mm was significantly correlated with CRT. As the CRT increases, the inner retinal layers are more compactly arranged and there is continuity of the INL, which therefore corresponds to a higher VFD in the central 1 mm in these eyes. Similarly, when the thickness decreases, the inner retinal layers are less compact and there is discontinuity of the INL, which corresponds to a lower VFD (Fig. 5).

In our study, there was a negative correlation between spherical error and VFD in the parafoveal region of eyes with myopia. Furthermore, compared to normal subjects the VFD was significantly lower in the parafoveal region of DRP of subjects with myopia. Similar to our study, Wang et al., did not find any significant difference between VFD in the SRP between myopics and emmetropics [19]. In another study, retinal layers of the macular region of subjects with myopia were segmented and compared to emmetropic subjects [20]. The results of the study demonstrated thinning of INL in patients with myopia and thickening of the Henle fiber layer and outer nuclear layer, myeloid and ellipsoid zone and outer segment of photoreceptors. This can explain the significant decrease in VFD we noted at the level of DRP, which is located at level of INL. We speculate that the decrease in retinal VFD at level of DRP can be one of the factors for retinal thinning of this region. However, we were unable to detect any difference in retinal thickness in subjects with myopia compared to normal. This can be because there is concomitant thickening and thinning of different retinal layers in myopics. Therefore, overall the change in total retinal thickness is small which was not detectable because of our small sample size.

The morphology and size of FAZ has been an area of interest in multiple studies. Samara et al. [21] extensively described the morphology of FAZ imaged using OCTA. Similar to what was noted in our study, shape of the FAZ was slightly irregular in all the eyes and the size of FAZ was larger and more irregular in the DRP compared to the SRP. They used ImageJ software to manually demarcate and measure the area of FAZ. This can be the reason that the mean area of FAZ noted in their study was slightly smaller in the superficial plexus (0.26 mm^2^) compared to our results (0.33 mm^2^). The size of the FAZ at level of DRP noted in their study was 0.46 mm^2^ which was very similar to what we noted (0.47 mm^2^). They also demonstrated an inverse correlation between area of FAZ and CRT. In our study we compared the area of FAZ and VFD in the central 1 mm and they were found to be inversely related. Both of the findings can be explained by the fact that as the CRT increases, the inner retinal layers are more compactly arranged and there is continuity of the INL in the foveal region (Fig. 5). Gade et all, in their algorithm demarcated FAZ with a continuous circle. [15] This means their FAZ measurements included some marginal vessels which would fall within that defined circle. Hence expectedly the mean FAZ area noted by them was marginally larger in both SRP (0.35 mm^2^) and DRP (0.49 mm^2^) compared to our study (0.33 and 0.47 mm^2^ respectively).

OCTA provides an effective way to visualize the vessels in the peripapillary region. This allows us to comment on their changes in morphology and VFD in various conditions. Liu et al. [22] demonstrated that peripapillary VFD is significantly decreased in glaucomatous eyes compared to the normal subjects (p < 0.001). Hollo et al. [23] measured VFD in superotemporal, temporal and inferotemporal peripapillary sectors and found a significant decrease in VFD in superotemporal and inferotemporal areas in glaucomatous eyes. Wang et al. [19] demonstrated a negative correlation between peripapillary vessel density and the grade of myopia. All of these studies underscore the value of measuring the VFD in peripapillary region. However, none of these studies reported VFD in the all the six sectors of the Garway-Heath map as reported in our study. Utilizing such a map would effectively allow us to measure changes in VFD in disease affecting the peripapillary region in each of sector and correlate the findings to the changes in RNFL thicknesses in each sector. In our study, the mean VFD in the peripapillary region was 60.4% (±5.0%). We did not any significant correlation between the VFD and RNFL thickness in normal population which can be secondary to our small sample size.

Even though we utilized standardized sector maps to measure VFDs via automated tools, potential limitations of our study included a small sample size. In addition, the measurement tool does not allow us to manually correct the measurements of FAZ, this may lead to errors in detection of FAZ boundaries in cases where images of low quality are analyzed. Our VFD measurements were limited to the SRP and DRP as we utilized the automated VFD measuring tool of the software which doesn’t automatically segment the intermediate capillary plexus of the retina.

## Conclusion

In conclusion, OCTA is an emerging noninvasive tool for imaging and quantifying retinal vasculature at the level both SRP and DRP and areas of non-perfusion such as FAZ. Utilizing standard sectors to measure VFD in macular and peripapillary region allows application of a uniform method to measure VFD in multiple conditions. Hence, VFD can be utilized as a standard outcome measure to monitor macular diseases and assess the effect of various therapies used for their management. In normal subjects, we found that the VFD in DRP is significantly higher than the superficial retinal plexuses, and VFD in the foveal region strongly correlates with central retinal thickness. Retinal thinning at level of INL in myopia may also be explained by a decrease in retinal VFD in the region.

## Abbreviations

CRT: central retinal thickness
DRP: deep retinal plexus
ETDRS: Early Treatment of Diabetic Retinopathy Severity
FAZ: foveal avascular zone
ILM: internal limiting membrane
IPL: inner plexiform layer
IRP: intermediate retinal plexus
OCTA: optical coherence tomography angiography
RPC: radial peripapillary capillaries
SRP: superficial retinal plexus
VFD: vessel flow density
